# Consumed by Boredom: Food Choice Motivation and Weight Changes during the COVID-19 Pandemic

**DOI:** 10.3390/bs12100366

**Published:** 2022-09-28

**Authors:** Wijnand A. P. Van Tilburg, Reinhard Pekrun, Eric R. Igou

**Affiliations:** 1 Department of Psychology, University of Essex, Colchester CO4 3SQ, UK; 2 Institute for Positive Psychology and Education, Australian Catholic University, North Sydney, NSW 2060, Australia; 3 Department of Psychology, University of Munich, 80802 Munich, Germany; 4 Department of Psychology, University of Limerick, V94 T9PX Limerick, Ireland

**Keywords:** boredom, eating motivation, weight, COVID-19, pandemic, health

## Abstract

Boredom is an established cause and correlate of eating behavior. Yet, existing work offers a scattered range of plausible motivations for why this is. We examined among 302 people representative of the adult UK population what motivations they had for selecting food during the COVID-19 pandemic and how this related to boredom. As predicted, bored people choose food less for health reasons and more for convenience. Boredom reduced ethical and ‘natural content’ motivations for selecting food and was not associated with choosing food to regulate one’s mood or to experience unfamiliarity. Boredom was also associated with greater absolute changes in weight over the course of the pandemic. Boredom did not predict weight gains or losses overall. These findings offer insights into the role that boredom plays in eating motivations in particular and health-relevant outcomes in general.

## 1. Introduction 

Boredom is a common unpleasant experience [1,2] often characterized as “the aversive experience of wanting, but being unable, to engage in satisfying activity” [3] (p. 482). The momentary experience of boredom involves a lack of perceived meaning [4,5] and either too little or too great challenge [6,7,8,9]. Those who are momentarily bored find time to be progressing slowly [7], experience attention failures [10], and let their mind wander [11]. People who feel bored tend to self-report low arousal [12,13], though physiological evidence suggests that whether arousal is low or high may depend on how it is measured [14]. At the motivational level, momentary boredom has been suggested to signal the need to get on with something more interesting, satisfying, novel, or meaningful [15,16,17,18,19,20,21]. Its set of reasonably consistent cognitive, motivational, and affective characteristics sets boredom apart from other negative affective states, such as anhedonia, depression, sadness, anger, frustration and others [9,13,22], and have led some to consider it a distinct emotion [13,23].

Boredom has been identified as a reason for people to eat [24,25,26], and it appears to motivate eating above and beyond the impact of other forms of negative affect [27,28]. Boredom is also a delinquent in the obesity epidemic [29] potential cause of both weight change [30,31], eating disorders [32,33], and is increasingly being accused of perpetrating poor physical health in general [34,35,36]. Yet, despite the established link between boredom and eating behavior, and despite the corresponding relevance of this link to physical and mental health, the evidence for alleged motivations that relate boredom to eating is surprisingly scattered. Some have found that boredom is associated with eating to improve mood [37]. Others have discovered that boredom involves eating food that satisfies sensation seeking [28]. Work that links boredom to issues such as impulsiveness and lack of self-control [38,39] hints that boredom may also encourage eating foods that are convenient to get; and research demonstrates that boredom encourages novelty-seeking [40,41] may point to boredom as a tentative source of craving unfamiliar foods, just to name a few.

What motivates people to eat out of boredom? Additionally, what role do these motivations play in weight gain or change among bored individuals? We examined these questions in the context of the recent COVID-19 pandemic, which may serve as psychological Petri dish for studying boredom and its consequences. These are important issues to address both theoretically and practically. Theories of boredom have proposed a large number of ways in which boredom affects motivation, ranging from sensation seeking [42] and a desire for novelty [40], to impulsiveness and loss of self-control [43,44]. Eating behavior offers a rare context that allows for a variety of motivations to be examined simultaneously (e.g., choosing food to satisfy sensation seeking, a desire for novelty, or an impulsive urge [45]).

The study of boredom and eating motivations can thus help understand how boredom relates to motivations matched in context, affording greater comparability. Furthermore, boredom allegedly plays an important role in perpetrating ill health and obesity [29]. Understanding *why* boredom may contribute to poor eating behavior can be critical for addressing this undesirable role. Only if practitioners and policymakers understand why boredom causes unhealthy eating can these underlying processes be targeted with effective interventions.

### 1.1. Boredom and the COVID-19 Pandemic

The COVID-19 pandemic, and its lockdowns, in particular, have arguably caused boredom to become a challenge for many, with suddenly limited opportunities to alleviate its occurrence [46,47,48,49]. Fitting the pandemic context, boredom occurs in response to monotony and repetitiveness [50]. Boredom surfaces in situations where one has little autonomy [51] and where one either lacks control or has too much of it [18]. In addition, research shows that loneliness—an issue that intensified during the COVID-19 pandemic [52]—is associated with elevated boredom proneness [1]. Furthermore, boredom affects people across ages and genders, albeit young adults and men are comparatively more likely to suffer it [2]. Thus, the pandemic may have festered boredom for many.

Indeed, studies show that many people experienced boredom during the pandemic (e.g., due to lockdowns) and with considerable intensity [48,49]. Boredom experienced during the pandemic affected people’s Bennetthealth-related behavior in several ways, including undermining commitment to safety precautions [46] and rousing political disagreement with health regulations [47,53]. Thus, boredom may have been particularly relevant to people’s health-related behavior, cognition, and motivations during this period. 

### 1.2. Boredom and Eating

Parallel to the rise of boredom during the COVID-19 pandemic, scholars have raised concerns about poorer eating behaviors during the same period [54]. Studies show that people reported eating-related challenges during the COVID-19 pandemic. For example, [55] raised eating-related concerns in light of availability and restricted access. Some studies suggest that people generally ate less healthily during the COVID-19 pandemic [56,57], which may have affected health-related outcomes such as people’s weight [58].

It is plausible that boredom played a role in diet and weight gain during the pandemic. Prior to it, the World Health Organization already accused boredom as one of the factors underlying the obesity epidemic [29]. Individuals who are particularly prone to boredom on average have a higher overall body-mass-index and boredom appears to undermine physical activity and exercise [36,59,60]. Furthermore, boredom may cause people to eat more or more unhealthily [26]. Indeed, boredom has been identified as a reason to eat among obese individuals [27]. Studies also show that people who suffer from an eating disorder report boredom as a reason for their eating [33]. Koball and colleagues [27] found that people blamed boredom above and beyond other negative affective states as a reason to eat. Consistently, in a diary study, Moynihan and colleagues [28] found that people tended to consume more calories, fats, carbohydrates, and proteins (but not fibers) on days that featured comparatively high levels of boredom. Furthermore, Moynihan et al. report that participants in an experiment were more likely to snack on unhealthy and exciting foods when watching an extremely dull versus comparatively engaging video. In sum, boredom plays a role in eating behavior. With the COVID-19 pandemic aggravating boredom, it is thus likely that boredom shaped eating behavior over this period. An examination of the link between boredom and different food motivations is warranted.

### 1.3. Current Study

We examined the link between boredom and people’s motivations for selecting specific types of food during the COVID-19 pandemic. Rather than focusing on a single eating motive (e.g., emotional coping), we instead assessed several tentative motivations, aided by the classification of food choice motives by Steptoe et al. [45]. Their corresponding food choice questionnaire is a self-report measure that assesses people’s motivation to select food based on nine key motives, featuring ones that have been suggested to link to boredom as well as others.

The first food choice motivation is selecting food based on health motives. We hypothesized that boredom would undermine health motivation. We anticipated so given that boredom correlates with impulsiveness [44], which is, in turn, a correlate of poor health decisions [61]. Furthermore, both boredom and poorer health decisions correlate with risk-taking [43,62], and boredom reduces health-promoting behaviors such as exercise [60]. The second food motivation features eating to improve mood. Several studies suggest that boredom, at least individual differences in boredom proneness, correlates with emotional eating [27,63]. Accordingly, we hypothesized a positive association between boredom and the motivation to eat food to improve one’s mood. The third food motivation was selecting food that is convenient in terms of being easily accessible. Based on boredom’s well-established relation with increased impulsiveness [42], reduced self-control [64], and accelerated temporal discounting [44], we hypothesized a positive association between boredom and convenience motivation. Selecting food that appeals to the senses was the fourth food motivation. Sensation seeking in general [42,65], and excitement seeking in food [28], are associated with boredom. We therefore hypothesized a positive association between boredom and the sensation seeking motive in food choices. The fifth food choice motivation concerns choosing food for its familiarity. Research suggests that individual differences in boredom proneness come with increased curiosity [66], and Bench and Lench [40] found that boredom encourages novelty seeking. Accordingly, we hypothesized that boredom would be negatively associated with the motivation to select food based on its familiarity. In addition to the five motivations above, Steptoe et al.’s [45] classification distinguishes motivations based on natural content, price, weight control, and ethical concerns. We did not have hypotheses for the link between these and boredom, and included them for exploratory purposes.

The link between boredom and food choice motivations was the primary focus of our investigation. Nonetheless, we also assessed and examined people’s weight gains and absolute change in weight. We had several reasons to do so. Firstly, doing so allowed us to further understand boredom’s alleged role in the obesity epidemic. Specifically, including (self-reported) weight measures allowed us to test which eating motivation(s) link boredom to weight changes. Secondly, researchers and health officials have expressed concerns about weight changes, especially in the context of the COVID-19 pandemic (e.g., [55,58]). Thus, including measures of weight allowed us to test the putative role of boredom and eating motives in relation to these concerns. Note that we examined both the link between boredom and absolute weight change as well as weight gain, in keeping with recent work on weight changes during the pandemic [58]. 

## 2. Method

### 2.1. Participants and Design

Participants were 302 adults from the general UK population. They were recruited through the high-quality [67] online crowdsourcing platform *Prolific.co* as a representative sample in age, gender, and ethnicity, based on UK Census estimates. Participants were paid GBP £1.50 (approx. USD $1.80) for the 10-min study. Four participants were excluded for completing the study faster than one-third of the median completion time or slower than three times the median completion time [68]. The final sample consisted of 298 participants (148 women, 144 men, 2 nonbinary, 4 unreported; *M*_age_ = 46.29, *SD*_age_ = 15.03, *Range* = 18–76). The study was correlational in design and received approval from the University of Essex’ ethical review board (ref: ETH2122-1155).

### 2.2. Materials and Procedure

The study took place online in May 2022. All participants first consented to taking part. Then, they reported how bored they felt over the course of the COVID-19 pandemic, using seven-point interval scales (1 = *not at all*, 7 = *very much*). Specifically, we asked them to “Please indicate how [bored] you generally felt from March 2020 to recent weeks, during which many societies implemented COVID-19 related restrictions.” We embedded this measure of boredom among other forms of affect to avoid demand effects. These other emotions included anger, shame, depression, disappointment, disgust, fear, frustration, guilt, happiness, pride, regret, and sadness. The presentation order of these emotions was random for each participant.

Subsequently, participants completed a 13-item measure of control and value appraisals [49,69,70]. Control was measured with seven items (e.g., “During the COVID-19 pandemic, I had a great deal of control over the important things in my life”; α = 0.69), and value was measured with six items (e.g., “My activities during the COVID-19 pandemic were useful for me”; α = 0.93), all rated on a five-point interval scale (1 = *Not at all true*, 5 = *Completely true*). (This measure was not further considered in light of the food motivation and weight, but the reader may be interested that control and value correlated negatively with boredom (*r* = −0.232, *p* < 0.001; *r* = −0.337, *p* < 0.001, respectively), consistent with control-value theory [69].)

The food choice motivation questionnaire [45] featured next. This self-report questionnaire contains 36 items that feature 9 distinct food choice motivations (health, mood, convenience, sensory appeal, natural content, price, weight control, familiarity, ethical concerns; αs ≥ 0.66). The instructions, slightly modified from the original to fit the COVID-19 context, read as follows:

*“Several different factors influence our choice of food. For every person, there will be a different set of factors that is important. In the next set of questions, we are interested in finding out what factors influenced your choice of food during the COVID-19 pandemic. Listed below are a series of factors that may be relevant to your choice of foods. Read each item carefully and decide how important the item is to you. Put a tick in the box that best reflects your feelings. Remember, there are no right or wrong answers—we are interested in what is important to you.”*

Participants then rated each of the 36 items with the stem “It was important to me that the food I ate on a typical day during the COVID-19 pandemic” (e.g., “tasted good”, “contained no additives”, “was low in calories”) on a five-point interval scale (1 = *Not important at all*, 5 = *Extremely important*).

Participants then reported gender, age, current employment status as well as employment status at the start of the pandemic (e.g., full time employment, part time employment, in school/university), living situation (e.g., with children 0–5 years in age, with parents, with partner), self-reported current weight and weight in early 2020, and measurement unit (all weights were converted into kg).

Participants next stated if there were reasons for their weight change (if there was one) on eight items. Specifically, we asked them, “If you gained or lost weight, what were the reasons? (leave blank if you did not gain or lose weight, or if you prefer not to say)”. We assessed weight change due to self-care (“Because I took better care of myself”, “Because I took better care of myself” (reversed), *r* = 0.592, *p* < 0.001), due to physical activity (“Because I became more physically active”, “Because I became less physically active” (reversed), *r* = 0.675, *p* < 0.001), due to negative emotions (“As a result of negative emotions”, “As a result of positive emotions” (reversed), *r* = 0.327, *p* < 0.001), and due to reduced availability (“Because there were fewer types of food available to me”, “Because there were more types of food available to me” (reversed), *r* = 0.081, *p* = 0.233). Participants were thanked and rewarded for their participation.

## 3. Results

### 3.1. Boredom during COVID-19 Pandemic in a Representative UK Sample

Our representative sample data indicated that boredom featured in the population prominently (*Median* = 5.00, *Mean* = 3.89, *SD* = 1.82, on a 7-point scale), with its confidence intervals placing it comfortably above the scale midpoint (95%*CI* = [3.69, 4.10], *SE* = 0.105). Our data did not indicate a significant difference between the experience of boredom among women (*M* = 3.84, *SD* = 1.181) versus men (*M* = 3.91, *SD* = 1.182), *t*(290) = 0.307, *p* = 0.759, *d* = 0.036, but did show that that boredom was more common among younger versus older participants, *r*(294) = −0.263, *p* < 0.001. Overall, boredom appears to have characterized people’s experience during the COVID-19 pandemic to a considerable degree, especially younger individuals.

### 3.2. Boredom and Food Choice Motivations during the COVID-19 Pandemic

Table 1 displays the zero-order correlations between boredom, food choice motivations, and weight changes. We predicted that boredom during the COVID-19 pandemic would be negatively associated with healthy food choice motivation and positively with choosing foods to improve one’s mood, for convenience, to offer sensory appeal, and due to unfamiliarity. We did not make predictions regarding motivations to select food that is perceived as ‘natural’, affordable, aids weight control, or satisfies ethical expectations.

Correlational analyses offered partial support for these predictions: Individuals who faced more boredom were significantly less motivated to choose healthy foods, *r*(298) = −0.129, *p* = 0.026, and were more motivated to select food based on convenience, *r*(298) = 0.123, *p* = 0.033. We did not find that bored individuals were motivated to consume food to regulate their mood, *r*(298) = −0.056, *p* = 0.337, or to select it on the basis of its (un)familiarity, *r*(298) = 0.039, *p* = 0.505. Interestingly, we found that those who felt more bored were marginally *less* inclined to select food on the basis of its sensory appeal, *r*(298) = −0.108, *p* = 0.062. We also found that boredom correlated significantly negatively with the motivation to choose food based on its ethical, *r*(298) = −0.119, *p* = 0.040, or natural, *r*(298) = −0.206, *p* < 0.001, content. 

### 3.3. Weight Changes during COVID-19 Pandemic

Of our 298 participants, 232 reported both their pre- and post-pandemic weights. We computed weight change for these individuals (in kilograms). Participants gained on average 0.92 kg (*SD*= 7.76, 95%*CI* = [−0.086, 1.922], *SE* = 0.510), with a median reported gain of 0 kg. While the overall weight gain was thus not significantly different from zero, we found that *absolute* weight did, in fact, change considerably, with an average (absolute) change of 5.01 kg (*SD* = 5.99, *Median* = 3.18, 95%*CI* = [4.232, 5.782], *SE* = 0.393). As for gender differences, neither weight gain (*M*_women_ = 1.07 kg, *SD* = 8.04, vs. *M*_men_ = 0.66 kg, *SD* = 7.58) nor absolute change in weight (*M*_women_ = 5.14 kg, *SD* = 6.25, vs. *M*_men_ = 4.88 kg, *SD* = 5.82) varied significantly, *t*(227) = 0.400, *p* = 0.690, *d* = 0.053, and, *t*(227) = 0.324, *p* = 0.746, *d* = 0.043. Age did not correlate significantly with weight gain, *r*(230) = −0.018, *p* = 0.790, but we did find that those of younger age reported higher rates of absolute change in weight, *r*(232) = −0.270, *p* < 0.001. In all, the average person’s weight changed by approximately 5 kg, representing a mix of gains and losses. This change in weight was greater among younger and lesser among older individuals. 

Boredom was not significantly correlated with weight gain, *r*(232) = 0.107, *p* = 0.104. However, we did find a significant correlation between boredom and absolute change in weight, *r*(232) = 0.186, *p* = 0.004, indicating that people who experienced more boredom during the COVID-19 pandemic faced greater changes in weight over that period.

Weight gain was significantly correlated with three of the food choice motivations: Those who gained weight reported significantly lower motivation to make healthy food choices, *r*(232) = −0.296, *p* < 0.001, selected food with lower ‘natural’ content, *r*(232) = −0.219, *p* < 0.001, and chose their food less on the basis of weight control considerations, *r*(232) = −0.309, *p* < 0.001. Weight gain did not correlate with the other motivations (*p*s ≥ 0.163). People’s absolute change in weight was significantly greater when they were motivated to choose food that helped regulate their mood, *r*(232) = 0.166, *p* = 0.011, and for sensory appeal, *r*(232) = 0.132, *p* = 0.044. Absolute changes in weight were smaller when accompanied by motivations to select food based on its healthiness, *r*(232) = −0.170, *p* = 0.010, and ‘natural’ content, *r*(232) = −0.141, *p* = 0.032. There were marginal associations between absolute weight change and convenience, *r*(232) = 0.115, *p* = 0.081, and food price, *r*(232) = 0.116, *p* = 0.078, motives. Absolute change in weight did not correlate with ethical, weight control, affordability, or ethical motivations (*p*s ≥ 0.100).

### 3.4. Motivations Linking Boredom to Weight

We next examined the link between boredom and weight changes in more detail; we probed the putative roles of food choice motivations and the attributions people made for their changes in weight (Table 2). Note that we examined this for both weight gains and absolute changes in weight, despite not finding a total association between boredom and the former. We did this in light of the fact that indirect associations may still exist even if a total association is absent, for example, when there are multiple opposed indirect associations [71].

**Indirect associations for weight gain.** Both boredom and weight gain related significantly to (reduced) healthy food choice motivation and (reduced) natural food choice motivation. These two motivations thus served as our candidate mediators to test if boredom was indirectly related to weight gain. We used the Process macro by Hayes [72] (model 4) to estimate their indirect effects using 5000 bias-corrected and accelerated bootstraps. We tested these indirect effects in separate mediation models.

Our first mediation analysis revealed a significantly positive indirect association between boredom and weight gain through reduced motivation to choose food that is healthy, *B* = 0.169, *SE* = 0.096, 95%*CI* = [0.0003, 0.3769]. Boredom predicted a reduction in motivation to choose healthy food, *B* = −0.070, *SE* = 0.034, *t*(230) = 2.064, *p* = 0.040, 95%*CI* = [−0.136, −0.0032], and the motivation to choose food for health reasons predicted, controlling for boredom, lower weight gain, *B* = −2.432, *SE* = 0.538, *t*(229) = 4.514, *p* < 0.001, 95%*CI* = [−3.4932, −1.3703] (Table 2, Mediation 1).

The second mediation analysis showed that the indirect association between boredom and weight gain through the motivation to choose ‘natural’ food was also significantly positive, *B* = 0.203, *SE* = 0.084, 95%*CI* = [0.0595, 0.3839]. Boredom undermined the motivation to choose food for its ‘natural’ content, *B* = −0.132, *SE* = 0.037, *t*(230) = 3.522, *p* < 0.001, 95%*CI* = [−0.2054, −0.0580]. This motivation predicted, in turn and controlling for boredom, significantly lower weight gain, *B* = −1.543, *SE* = 0.497, *t*(229) = 3.108, *p* = 0.002, 95%*CI* = [−2.5214, −0.5646] (Table 2, Mediation 2). 

**Indirect associations for absolute weight change.** Prior analyses established that boredom and absolute weight change correlated significantly negatively with the motivations to select healthy food and natural content food. Furthermore, boredom correlated with marginally *less* food choices based on sensory appeal whereas absolute weight change correlated positively with that. Accordingly, we tested mediational models where healthy food, natural content, sensory appeal choice motivations featured as mediators of the association between boredom and absolute changes in weight. We again used the Process macro by Hayes [72] (model 4) with 5000 bias-corrected and accelerated bootstraps. 

Our first mediation analysis revealed that the motivation to select healthy food marginally mediated the association between boredom and absolute weight change, *B* = 0.067, *SE* = 0.052, 95%*CI* = [−0.0059, 0.1899]. Boredom again predicted a reduction in motivation to choose healthy food, *B* = −0.070, *SE* = 0.034, *t*(230) = 2.064, *p* = 0.040, 95%*CI* = [−0.1306, −0.0032], and, controlling for boredom, the motivation to choose food for health reasons predicted, lower absolute weight change, *B* = −0.965, *SE* = 0.424, *t*(229) = 2.274, *p* = 0.024, 95%*CI* = [−1.800, −0.1288] (Table 2, Mediation 3).

The second mediation analysis showed that the motivation to choose food for its sensory appeal significantly mediated the association between boredom and absolute weight change, *B* = −0.103, *SE* = 0.055, 95%*CI* = [−0.2266, −0.0143]. Boredom predicted a *reduced* motivation to opt for foods that appeal to the senses, *B* = −0.070, *SE* = 0.026, *t*(230) = 2.712, *p* = 0.007, 95%*CI* = [−0.1210, −0.0192]; the motivation to choose food for sensory appeal also still predicted higher absolute weight change, when controlling for boredom, *B* = 1.446, *SE* = 0.552, *t*(229) = 2.621, *p* = 0.009, 95%*CI* = [0.3589, 2.5329] (Table 2, Mediation 4).

A third mediation analysis, featuring natural content motivation as mediator, showed that this variable did not significantly mediate the association between boredom and absolute weight change, *B* = 0.079, *SE* = 0.055, 95%*CI* = [−0.0166, 0.1960]. Boredom predicted a reduced motivation to opt for ‘natural’ foods, *B* = −0.132, *SE* = 0.037, *t*(230) = 3.522, *p* < 0.001, 95%*CI* = [−0.2054, −0.0580]; the motivation to choose natural food no longer related significantly to higher absolute weight change, when controlling for boredom, *B* = −0.603, *SE* = 0.385, *t*(229) = 1.566, *p* = 0.119, 95%*CI* = [−1.3605, 0.1555] (Table 2, Mediation 5).

## 4. Discussion

We examined how boredom related to food choice motivations during the COVID-19 pandemic. While prior work established links between boredom and eating [24,25,26,27,28], the motivations underlying this link were poorly understood. We addressed this issue by utilizing a measure of multiple food motivations [45] and correlating boredom with each. Importantly, this study examined people’s self-reported boredom and food choice motivations over the course of the COVID-19 pandemic, which is a particularly relevant period. Boredom proved a challenge for many over the course of this period, and several studies indicate that boredom aggravated poor health choices and safety behavior [46,47,48]. At the same time, experts expressed concerns about dietary behavior during the pandemic and weight changes [55,56,57,58]. In light of boredom’s link to eating behavior, it is thus pertinent to study how boredom related to food choice motivations during the COVID-19 pandemic.

Our results confirm that the experience of boredom was indeed prominent, especially among younger people, during the pandemic. Furthermore, we found that people’s boredom related to a number of motivations for choosing foods. As predicted, we found that those whose pandemic experience was characterized more strongly by boredom were less motivated to select healthy foods and were more likely to choose foods based on convenience. These findings are consistent with prior observations showing boredom as a cause or correlate of impulsiveness, riskier [43] or more arbitrary [73] decision profiles, reduced self-control [64], and accelerated temporal discounting [44]. We found that boredom’s association with reduced health considerations in food choices, in turn, related it to weight gains as part of an indirect association. While boredom did not feature weight gain overall, the results suggest that it likely contributed to it indirectly by undermining the motivation to choose healthy foods.

Contrary to our predictions, we did not find that boredom was associated with choosing foods to regulate one’s mood. This finding seems to go against some prior work that identified emotional coping as a reason why boredom relates to eating. It may well be, however, that there are important moderators to consider in this association. For example, Ferrell and colleagues [74] found that working memory capacity qualifies the links between boredom, emotional eating, and emotion regulation difficulties. In addition, we did not find that bored people were motivated to try unfamiliar foods. This finding suggests that the curiosity and novelty-seeking typically associated with boredom [40,66] may either not apply to eating behavior, may be counteracted by convenience motives, or, possibly, may have been curtailed by reduced availability of novel food experiences during the pandemic.

While we did not make predictions regarding the links between boredom on the one hand and choosing foods for ethical or ‘natural’ content on the other, we found, interestingly, that boredom was associated with lower motivations for both. Perhaps this unexpected finding can be attributed to the convenience that bored people seek in the food they choose. After all, selecting food on the basis of its convenience plausibly undermines the effortful consideration of the ethical footprint of the food or its perceived natural content. Intriguingly, the reduced motivation among bored people to consider ‘natural’ food content as part of their food choices mediated the association between boredom and absolute weight changes.

A striking finding was that those whose pandemic experience was more strongly characterized by boredom reported a marginally *lesser* motivation to choose foods based on sensory appeal. We had predicted precisely the opposite. Existing studies indicate that boredom features sensation seeking [42,65], and experimental work has shown that boredom can even promote eating healthy food provided that it appeals to the senses [28]. One possibility is that, again, the motivation to select convenient food under boredom undermines selecting food that appeals to the senses. Given that the current finding is opposite to the association we predicted, though, it is particularly important that this inconsistency is resolved empirically in future research, even more so as our data indicated that this motivation partially mediated the association between boredom and changes in absolute weight.

### Contributions, Limitations, and Future Directions

Our examination of boredom featured a rather large number of tentative food choice motivations it may relate to. This variety in potential motivational processes associated with boredom mirrors the heterogeneity in processes ascribed to boredom in contemporary models. Eastwood and Gorelick’s [75] model of unused cognitive potential, for example, suggests that boredom plays a pivotal role in regulating cognitive engagement. Van Tilburg and Igou [4,5,38] propose as part of their pragmatic meaning regulation account that boredom instigates a search for more meaningful activities (e.g., those that are perceived as instrumental in the pursuit of valued outcomes; [76]), in addition to seeking challenge [9]. The meaning and attention model of boredom by Westgate and Wilson [21] proposes that momentarily bored people look for more interesting or enjoyable things, depending on whether their boredom features low attention, low meaning, or both. In their boredom feedback model, Tam and colleagues [20] propose that boredom shifts attention outwards (e.g., novel activities), inwards (e.g., mind-wandering), or back to an activity at hand (e.g., reappraisal) with the ultimate goal of optimizing attentional engagement. Pekrun’s control-value theory [8,18,77,78] proposes that boredom experienced in achievement settings undermines current achievement activities but can steer an individual to more productive and worthwhile alternative actions [79]. Consistent with these self-regulatory propositions, Bench and Lench [40] argue that boredom regulates behavior in the pursuit of novel experiences (whether pleasant or not). Elpidorou [16] proposes that boredom regulates satisfactory goal pursuit more generally, and Danckert [80] suggests that boredom encourages attempts to have an impact on one’s environment (i.e., effectance). Model specifics aside, each of these recent accounts of boredom agrees that this experience serves to regulate people’s behavior or cognition; it is a functional psychological state, and, in being so, offers a window into corresponding motivations.

Accepting the assumption that boredom serves self-regulation, we are subsequently left with a rather unwieldy list of phenomena allegedly being regulated, including challenge, meaning, attention, interest, enjoyment, satisfaction, novelty, efficacy, and cognitive engagement. On the one hand, this theoretical heterogeneity—plausibly a symptom of the recent surge in boredom research—calls (screams?) for attempts at integration and parsimony. On the other hand, it offers a wealth of putative mechanisms that may help us understand boredom’s link(s) with its consequences. It is this latter approach that we adopted in the present investigation, where we attempt to unravel boredom’s link to eating motivation. However, we recognize the importance of the former and encourage researchers to examine unifying principles that can help reduce the unwieldy number of processes allegedly regulated by boredom into a key few.

The correlates and consequences of boredom are considerably varied in content, and many are notoriously problematic. For example, the momentary experience of boredom can cause sadism [81], intergroup bias [4], reduced academic performance [18], risk-taking or noisy decision-making [43,73], and counterproductive work behavior [82], to name a few. Furthermore, on average, individuals who are prone to boredom suffer challenges in well-being (e.g., lower life satisfaction, reduced meaning in life, stress, depression), self-regulation (e.g., impaired self-control, impulsiveness, reduced promotion and prevention foci), cognition (e.g., attention failures, inflexibility), motivation (e.g., loss of interest), and behavior (e.g., risk-taking, problematic phone use, binge drinking, procrastination, school dropout; for reviews, see [35,83,84]). Perhaps offering a faint light in darkness, boredom-prone individuals show elevated curiosity [66]. In addition, boredom seems to encourage original thinking [85], prosocial commitments [86], and self-soothing nostalgic reverie [87].

How do the current findings fit into this arsenal of prior findings? It is tempting to view boredom as a problematic influence on healthy eating motivations and ethical food considerations in lieu of food that is convenient. This tentatively problematic role in food choice motivation portrays boredom as an undesirable player when it comes to health-related behavior in general, alongside earlier work indicating that boredom undermines healthy exercise [18,49] and the suggestion that boredom may increase cardiovascular risks [88]. Research on the health implications of boredom is lacking, and we hope that the current findings offer another stepping stone in its dedicated study.

There is great variability in the methods of earlier work on boredom and eating behavior and motivations. For example, some [89,90] relied on qualitative interviews to identify the food motivations associated with boredom. Others correlated eating for emotional coping with individual differences in boredom proneness [37]. Yet, others relied on diary studies of daily boredom or experimental induction of momentary boredom [28]. These methodological differences are important as they might lead to different findings. The current study relied on cross-sectional self-reports, and it is possible that behavioral operationalizations would give different results, for example, where people’s perceptions and behaviors diverge. Likewise, the nature of our self-report measures required participants to consider and evaluate a relatively long period of time. Momentary assessment of boredom, for example, through diary study methods, may reveal patterns that the current methodology would have overlooked.

We examined the link between boredom and eating motivation in a UK sample. However, is possible that findings would have been different had we performed our study within another cultural setting or with samples from different ethnicities. While there are few comprehensive comparative studies on boredom, there is evidence that the experience of boredom may differ across cultural and ethnic contexts. Ng et al. (2015), for example, found that European Canadians reported higher levels of boredom than Chinese Canadians. Work by Vodanovich and colleagues (2011) showed that people in the USA find themselves more prone to boredom than people in Germany. We are not aware of dedicated studies that compare boredom in the UK against other societies, but if experienced levels of boredom are systematically lower or higher in the UK than elsewhere, then this would tell us more about the potential significance of boredom in eating motivation and weight change. Specifically, based on our finding that boredom is associated with greater changes in absolute weight, we can anticipate larger weight fluctuations in societies where boredom is relatively prominent. Corresponding comparative studies may focus on cultural factors such as beliefs about ideal affect, which Ng et al. (2015) propose is one key reason for differences between groups in the experience of boredom.

## 5. Conclusions

We studied how boredom related to food choice motivations over the course of the COVID-19 pandemic. Data from a representative UK adult sample indicate that boredom was a prominent experience for many people, especially for those who were younger. The findings suggest that those whose pandemic experience was characterized more strongly by boredom chose foods out of convenience and less for health, ethical, or ‘natural’ content reasons. Boredom was also associated with greater absolute changes in weight but not with a general increase or decrease. Our work demonstrates the significance of boredom for eating behavior in the context of the COVID-19 pandemic and points to convenience-eating as a key motivation. More generally, this work helps to establish further boredom’s position in relation to health-related outcomes.

## Figures and Tables

**Table 1:** Zero-order correlations.

		1	2	3	4	5	6	7	8	9	10	11	12
1	Boredom	-	−0.129 *	0.056	0.123 *	−0.108	−0.206 **	0.082	−0.025	0.039	−0.119 *	0.107	0.186 **
2	Health		-	0.192 **	0.033	0.248 **	0.704 **	0.059	0.552 **	0.118 *	0.494 **	−0.296 **	−0.170 **
3	Mood regulation			-	0.260 **	0.513 **	0.154 **	0.105	0.125	0.278 **	0.182 **	0.024	0.166 *
4	Convenience				-	0.183 **	−0.060	0.342 **	0.138	0.493 **	−0.008	−0.070	0.115
5	Sensory appeal					-	0.145 *	0.161 **	0.038	0.230 **	0.166 **	0.039	0.132 *
6	Natural content						-	−0.032	0.421	0.044	0.524 **	−0.219 **	−0.141 *
7	Price							-	0.083	0.212 **	0.086	−0.018	0.116
8	Weight control								-	0.129 *	0.359 **	−0.309 **	0.025
9	Familiarity									-	0.196 **	−0.021	0.104
10	Ethical concerns										-	−0.092	−0.005
11	Weight gain											-	0.233 **
12	Absolute weight change												-

Note: * *p* ≤ 0.05, ** *p* ≤ 0.01. Variables 2 through 10 represent food choice motivations.

**Table 2:** Mediation analyses.

Mediation	Predictor	Outcome	*B*	*SE*	*t*	*p*	95%*CI*
1	Boredom	Health	−0.070	0.034	20.064	0.040	[−0.1360,−0.0032]
1	Boredom	Weight gain	0.299	0.278	10.075	0.284	[−0.2490, 0.8467]
1	Health	Weight gain	−20.432	0.538	40.514	<0.001	[−30.4932, −10.3703]
2	Boredom	Natural content	−0.132	0.037	30.522	<0.001	[−0.2054, −0.0580]
2	Boredom	Weight gain	0.265	0.289	0.916	0.3361	[−0.3048, −0.8346]
2	Natural content	Weight gain	−10.543	0.497	30.108	0.002	[−20.5214, −0.5646]
3	Boredom	Health	−0.070	0.034	20.064	0.040	[−0.1360, −0.0032]
3	Boredom	Absolute weight change	0.562	0.219	20.567	0.011	[0.1306, 0.9933]
3	Health	Absolute weight change	−0.965	0.424	20.274	0.024	[−10.800, −0.1288]
4	Boredom	Sensory appeal	−0.070	0.026	20.712	0.007	[−0.1210, −0.0192]
4	Boredom	Absolute weight change	0.730	0.220	30.327	0.001	[0.2978, 10.1630]
4	Sensory appeal	Absolute weight change	10.446	0.552	20.621	0.009	[0.3589, 20.5329]
5	Boredom	Natural content	−0.132	0.037	30.522	<0.001	[−0.2054, −0.0580]
5	Boredom	Absolute weight change	0.550	0.224	20.454	0.015	[0.1084, 0.9911]
5	Natural content	Absolute weight change	−0.603	0.385	10.566	0.119	[−10.3605, 0.1555]

## Data Availability

The data presented in this study are openly available in Open Science Foundation at https://osf.io/3znt6/?view_only=b532e8a502c348ca83f4c861ae3e2d42.

## References

[1] Chan C.S., Van Tilburg W.A.P., Igou E.R., Poon C., Tam K.Y., Wong V.U., Cheung S.K. (2018). Situational meaninglessness and state boredom: Cross-sectional and experience-sampling findings. Motiv. Emot..

[2] Chin A., Markey A., Bhargava S., Kassam K.S., Loewenstein G. (2017). Bored in the USA: Experience sampling and boredom in everyday life. Emotion.

[3] Eastwood J.D., Frischen A., Fenske M.J., Smilek D. (2012). The unengaged mind: Defining boredom in terms of attention. Perspect. Psychol. Sci..

[4] Van Tilburg W.A.P., Igou E.R. (2011). On boredom and social identity: A pragmatic meaning-regulation approach. Personal. Soc. Psychol. Bull..

[5] Van Tilburg W.A.P., Igou E.R., Ros Velasco J. (2019). The unbearable lightness of boredom: A pragmatic meaning-regulation hypothesis. Boredom Is in Your Mind: A Shared Psychological-Philosophical Approach.

[6] Csikszentmihalyi M., Abuhamdeh S., Nakamura J. (2014). Flow. Flow and the Foundations of Positive Psychology.

[7] Danckert J.A., Allman A.A.A. (2005). Time flies when you’re having fun: Temporal estimation and the experience of boredom. Brain Cogn..

[8] Pekrun R. (2006). The control-value theory of achievement emotions: Assumptions, corollaries, and implications for educational research and practice. Educ. Psychol. Rev..

[9] Van Tilburg W.A.P., Igou E.R. (2012). On boredom: Lack of challenge and meaning as distinct boredom experiences. Motiv. Emot..

[10] Hunter A., Eastwood J.D. (2018). Does state boredom cause failures of attention? Examining the relations between trait boredom, state boredom, and sustained attention. Exp. Brain Res..

[11] Danckert J., Merrifield C. (2018). Boredom, sustained attention and the default mode network. Exp. Brain Res..

[12] Smith C.A., Ellsworth P.C. (1985). Patterns of cognitive appraisal in emotion. J. Personal. Soc. Psychol..

[13] Van Tilburg W.A.P., Igou E.R. (2017). Boredom begs to differ: Differentiation from other negative emotions. Emotion.

[14] Merrifield C., Danckert J. (2014). Characterizing the psychophysiological signature of boredom. Exp. Brain Res..

[15] Bench S.W., Lench H.C. (2013). On the function of boredom. Behav. Sci..

[16] Elpidorou A. (2018). The bored mind is a guiding mind: Toward a regulatory theory of boredom. Phenomenol. Cogn. Sci..

[17] Elpidorou A. (2022). Boredom and Poverty: A Theoretical Model. The Moral Psychology of Boredom.

[18] Pekrun R., Goetz T., Daniels L.M., Stupnisky R.H., Perry R.P. (2010). Boredom in achievement settings: Exploring control–value antecedents and performance outcomes of a neglected emotion. J. Educ. Psychol..

[19] Struk A.A., Scholer A.A., Danckert J. (2016). A self-regulatory approach to understanding boredom proneness. Cogn. Emot..

[20] Tam K.Y., Van Tilburg W.A.P., Chan C.S., Igou E.R., Lau H. (2021). Attention drifting in and out: The boredom feedback model. Personal. Soc. Psychol. Rev..

[21] Westgate E.C., Wilson T.D. (2018). Boring thoughts and bored minds: The MAC model of boredom and cognitive engagement. Psychol. Rev..

[22] Goldberg Y.K., Eastwood J.D., LaGuardia J., Danckert J. (2011). Boredom: An emotional experience distinct from apathy, anhedonia, or depression. J. Soc. Clin. Psychol..

[23] Pekrun R., Goetz T., Bieleke M., Wolff W., Martarelli C. (2022). Boredom: A control-value approach. The Routledge International Handbook of Boredom.

[24] Abramson E.E., Stinson S.G. (1977). Boredom and eating in obese and non-obese individuals. Addict. Behav..

[25] Havermans R.C., Vancleef L., Kalamatianos A., Nederkoorn C. (2015). Eating and inflicting pain out of boredom. Appetite.

[26] Jackson A., Anderson A., Weybright E., Lanigan J. (2021). Differing experiences of boredom during the pandemic and associations with dietary behaviors. J. Nutr. Educ. Behav..

[27] Koball A.M., Meers M.R., Storfer-Isser A., Domoff S.E., Musher-Eizenman D.R. (2012). Eating when bored: Revision of the emotional eating scale with a focus on boredom. Health Psychol..

[28] Moynihan A.B., Van Tilburg W.A.P., Igou E.R., Wisman A., Donnelly A.E., Mulcaire J.B. (2015). Eaten up by boredom: Consuming food to escape awareness of the bored self. Front. Psychol..

[29] World Health Organization-Europe (WHO) (2007). The Challenge of Obesity in the WHO European Region and the Strategies for Response. http://www.euro.who.int/__data/assets/pdf_file/0010/74746/E90711.pdf.

[30] Bhutani S., Cooper J.A. (2020). COVID-19–related home confinement in adults: Weight gain risks and opportunities. Obesity.

[31] Pellegrini M., Ponzo V., Rosato R., Scumaci E., Goitre I., Benso A., Pellegrini M., Ponzo V., Rosato R., Scumaci E. (2020). Changes in weight and nutritional habits in adults with obesity during the “lockdown” period caused by the COVID-19 virus emergency. Nutrients.

[32] Craparo G., Gagliano O., Costanzo G., La Rosa V.L., Gori A., Mendolicchio L. (2020). Boredom, alexithymia, and desire thinking in eating disorders: A cross-sectional study. Mediterr. J. Clin. Psychol..

[33] Wiedemann A.A., Ivezaj V., Barnes R.D. (2018). Characterizing emotional overeating among patients with and without binge-eating disorder in primary care. Gen. Hosp. Psychiatry.

[34] Sommers J., Vodanovich S.J. (2000). Boredom proneness: Its relationship to psychological-and physical-health symptoms. J. Clin. Psychol..

[35] Van Tilburg W.A.P., Moynihan A.B., Chan C.S., Igou E.R., Bieleke M., Wolff W., Martarelli C. (2022). Boredom proneness. The Routledge International Handbook of Boredom.

[36] Wolff W., Bieleke M., Martarelli C.S., Danckert J. (2021). A primer on the role of boredom in self-controlled sports and exercise behavior. Front. Psychol..

[37] Ahlich E., Rancourt D. (2022). Boredom proneness, interoception, and emotional eating. Appetite.

[38] Moynihan A.B., Igou E.R., Van Tilburg W.A.P. (2021). Existential escape of the bored: A review of meaning-regulation processes under boredom. Eur. Rev. Soc. Psychol..

[39] Tian Z.P., Liu Y., Yang K., Ma D.H. (2016). Effects of boredom proneness and state boredom on eating behavior: Mediating role of self-control. Chin. J. Clin. Psychol..

[40] Bench S.W., Lench H.C. (2019). Boredom as a seeking state: Boredom prompts the pursuit of novel (even negative) experiences. Emotion.

[41] Dalle Grave R., Calugi S., Marchesini G., Beck-Peccoz P., Bosello O., Compare A., Cuzzolaro M., Grossi E., Mannucci E., Molinari E. (2013). Personality features of obese women in relation to binge eating and night eating. Psychiatry Res..

[42] Dahlen E.R., Martin R.C., Ragan K., Kuhlman M.M. (2004). Boredom proneness in anger and aggression: Effects of impulsiveness and sensation seeking. Personal. Individ. Differ..

[43] Kılıç A., Van Tilburg W.A.P., Igou E.R. (2020). Risk-taking increases under boredom. J. Behav. Decis. Mak..

[44] Moynihan A.B., Igou E.R., Van Tilburg W.A.P. (2017). Boredom increases impulsiveness: A meaning-regulation perspective. Soc. Psychol..

[45] Steptoe A., Pollard T.M., Wardle J. (1995). Development of a measure of the motives underlying the selection of food: The food choice questionnaire. Appetite.

[46] Boylan J., Seli P., Scholer A.A., Danckert J. (2021). Boredom in the COVID-19 pandemic: Trait boredom proneness, the desire to act, and rule-breaking. Personal. Individ. Differ..

[47] Brosowsky N.P., Van Tilburg W.A.P., Scholer A.A., Boylan J., Seli P., Danckert J. (2021). Boredom proneness, political orientation and adherence to social-distancing in the pandemic. Motiv. Emot..

[48] Martarelli C.S., Wolff W., Bieleke M. (2021). Bored by bothering? A cost-value approach to pandemic boredom. Humanit. Soc. Sci. Commun..

[49] Pekrun R., Marsh H.W., Elliot A.J., Loderer K., Perry R.P., Vogl E., Goetz T., Van Tilburg W.A.P., Lüdtke O., Vispoel W. (2022). A three-dimensional taxonomy of achievement emotions. J. Personal. Soc. Psychol..

[50] Cummings M.L., Gao F., Thornburg K.M. (2016). Boredom in the workplace: A new look at an old problem. Hum. Factors.

[51] Van Hooff M.L., Van Hooft E.A. (2014). Boredom at work: Proximal and distal consequences of affective work-related boredom. J. Occup. Health Psychol..

[52] Palgi Y., Shrira A., Ring L., Bodner E., Avidor S., Bergman Y., Cohen-Fidel S., Keisari S., Hoffman Y. (2020). The loneliness pandemic: Loneliness and other concomitants of depression, anxiety and their comorbidity during the COVID-19 outbreak. J. Affect. Disord..

[53] Westgate E., Buttrick N., Lin Y., El Helou G.M., Agostini M., Belanger J., Gutzkow B., Kreienkamp J., Abakoumkin G., Abdul Khaiyom J.H.B. (2021). Pandemic Boredom: Little Evidence That Lockdown-Related Boredom Affects Risky Public Health Behaviors across 116 Countries. https://psyarxiv.com/78kma.

[54] González-Monroy C., Gómez-Gómez I., Olarte-Sánchez C.M., Motrico E. (2021). Eating behaviour changes during the COVID-19 pandemic: A systematic review of longitudinal studies. Int. J. Environ. Res. Public Health.

[55] Khan M.A., Smith J.E.M. (2020). “Covibesity,” a new pandemic. Obes. Med..

[56] Chatterjee P., Nirgude A., Chatterjee P.K. (2022). Healthy eating–a modifiable contributor to optimize healthy living in the COVID-19 pandemic: A review. J. Sci. Food Agric..

[57] Esobi I.C., Lasode M.K., Barriguete M.F. (2020). The impact of COVID-19 on healthy eating habits. J. Clin. Nutr. Health.

[58] Khan M.A., Menon P., Govender R., Samra A.M.A., Allaham K.K., Nauman J., Östlundh L., Mustafa H., Smith J.E.M., AlKaabi J.M. (2022). Systematic review of the effects of pandemic confinements on body weight and their determinants. Br. J. Nutr..

[59] Wolff W., Martarelli C.S. (2020). Bored into depletion? Toward a tentative integration of perceived self-control exertion and boredom as guiding signals for goal-directed behavior. Perspect. Psychol. Sci..

[60] Wolff W., Bieleke M., Stähler J., Schüler J. (2021). Too bored for sports? Adaptive and less-adaptive latent personality profiles for exercise behavior. Psychol. Sport Exerc..

[61] Van Beurden S.B., Greaves C.J., Smith J.R., Abraham C. (2016). Techniques for modifying impulsive processes associated with unhealthy eating: A systematic review. Health Psychol..

[62] Gough B., Conner M.T. (2006). Barriers to healthy eating amongst men: A qualitative analysis. Soc. Sci. Med..

[63] Braden A., Musher-Eizenman D., Watford T., Emley E. (2018). Eating when depressed, anxious, bored, or happy: Are emotional eating types associated with unique psychological and physical health correlates?. Appetite.

[64] Bieleke M., Barton L., Wolff W. (2021). Trajectories of boredom in self-control demanding tasks. Cogn. Emot..

[65] Kass S.J., Vodanovich S.J. (1990). Boredom proneness: Its relationship to Type A behavior pattern and sensation seeking. Psychol. J. Hum. Behav..

[66] Hunter J.A., Abraham E.H., Hunter A.G., Goldberg L.C., Eastwood J.D. (2016). Personality and boredom proneness in the prediction of creativity and curiosity. Think. Ski. Creat..

[67] Palan S., Schitter C. (2018). Prolific. ac—A subject pool for online experiments. J. Behav. Exp. Financ..

[68] Mahadevan N., Gregg A.P., Sedikides C. (2019). Is self-regard a sociometer or a hierometer? Self-esteem tracks status and inclusion, narcissism tracks status. J. Personal. Soc. Psychol..

[69] Perry R.P., Hladkyi S., Pekrun R., Pelletier S. (2001). Academic control and action control in college students: A longitudinal study of self-regulation. J. Educ. Psychol..

[70] Pekrun R., Vogl E., Muis K.R., Sinatra G.M. (2017). Measuring emotions during epistemic activities: The Epistemically-Related Emotion Scales. Cogn. Emot..

[71] Hayes A.F. (2009). Beyond Baron and Kenny: Statistical mediation analysis in the new millennium. Commun. Monogr..

[72] Hayes A.F. (2017). Introduction to Mediation, Moderation, and Conditional Process Analysis: A Regression-Based Approach.

[73] Yakobi O., Danckert J. (2021). Boredom proneness is associated with noisy decision-making, not risk-taking. Exp. Brain Res..

[74] Ferrell E.L., Watford T.S., Braden A. (2020). Emotion regulation difficulties and impaired working memory interact to predict boredom emotional eating. Appetite.

[75] Eastwood J.D., Gorelik D. (2019). Boredom is a feeling of thinking and a double-edged sword. Boredom Is in Your Mind.

[76] Van Tilburg W.A.P., Igou E.R. (2013). On the meaningfulness of behavior: An expectancy x value approach. Motiv. Emot..

[77] Pekrun R., Dicke T., Guay F., Marsh H.W., Craven R.G., McInerney D.M. (2021). Self-appraisals and emotions: A generalized control-value approach. Self—A Multidisciplinary Concept.

[78] Pekrun R., Perry R.P. (2014). Control-value theory of achievement emotions. International Handbook of Emotions in Education.

[79] Haager J.S., Kuhbandner C., Pekrun R. (2018). To be bored or not to be bored—How task-related boredom influences creative performance. J. Creat. Behav..

[80] Danckert J. (2019). Boredom: Managing the delicate balance between exploration and exploitation. Boredom Is in Your Mind.

[81] Pfattheicher S., Lazarević L.B., Westgate E.C., Schindler S. (2021). On the relation of boredom and sadistic aggression. J. Personal. Soc. Psychol..

[82] Van Hooft E.A., Van Hooff M.L. (2018). The state of boredom: Frustrating or depressing?. Motiv. Emot..

[83] Vodanovich S.J. (2003). Psychometric measures of boredom: A review of the literature. J. Psychol..

[84] Vodanovich S.J., Watt J.D. (2016). Self-report measures of boredom: An updated review of the literature. J. Psychol..

[85] Mann S., Cadman R. (2014). Does being bored make us more creative?. Creat. Res. J..

[86] Van Tilburg W.A.P., Igou E.R. (2017). Can boredom help? Increased prosocial intentions in response to boredom. Self Identity.

[87] Van Tilburg W.A.P., Igou E.R., Sedikides C. (2013). In search of meaningfulness: Nostalgia as an antidote to boredom. Emotion.

[88] Britton A., Shipley M.J. (2010). Bored to death?. Int. J. Epidemiol..

[89] Bennett J., Greene G., Schwartz-Barcott D. (2013). Perceptions of emotional eating behavior. A qualitative study of college students. Appetite.

[90] Pretlow R.A. (2011). Addiction to highly pleasurable food as a cause of the childhood obesity epidemic: A qualitative Internet study. Eat. Disord..

